# Supplementary material for: Tunable Thermo-Responsive Copolymers from DEGMA and OEGMA Synthesized by RAFT Polymerization and the Effect of the Concentration and Saline Phosphate Buffer on Its Phase Transition
Source: Polymers (Basel). 2019 Oct 11;11(10):1657. doi: 10.3390/polym11101657 (PMC6835898; doi:10.3390/polym11101657)
Supplement: Supplementary file 1 [file polymers-11-01657-s001.pdf]

Supplementary Materials

Article

Tunable Thermo-Responsive Copolymers from DEGMA and OEGMA Synthesized by RAFT Polymerization and the Effect of the Concentration and Saline Phosphate Buffer on its Phase Transition

Alejandro Ramírez-Jiménez [1,*], Kathleen Abigail Montoya-Villegas [2], Angel Licea-Claverie [2] and Mirian Angelene Gónzalez-Ayón [2]

[1] Cátedras CONACyT-Centro de Graduados e Investigación en Química, Tecnológico Nacional de México/Instituto Tecnológico de Tijuana, Blvd. Alberto Limón Padilla S/N, Ciudad Industrial Mesa de Otay, 22500 Tijuana, Baja California, México

[2] Centro de Graduados e Investigación en Química, Tecnológico Nacional de México/Instituto Tecnológico de Tijuana, Blvd. Alberto Limón Padilla S/N, Ciudad Industrial Mesa de Otay, 22500 Tijuana, Baja California, México. kathleen.montoya@tectijuana.edu.mx (K.A.M.-V); aliceac@tectijuana.mx (A.L.-C.); mirian.gonzalez@tectijuana.edu.mx (M.A.G.-A.)

* Correspondence: aramirezj@conacyt.mx; Tel.: +52-664-623-3772

Received: 28 August 2019; Accepted: 20 September 2019; Published: date

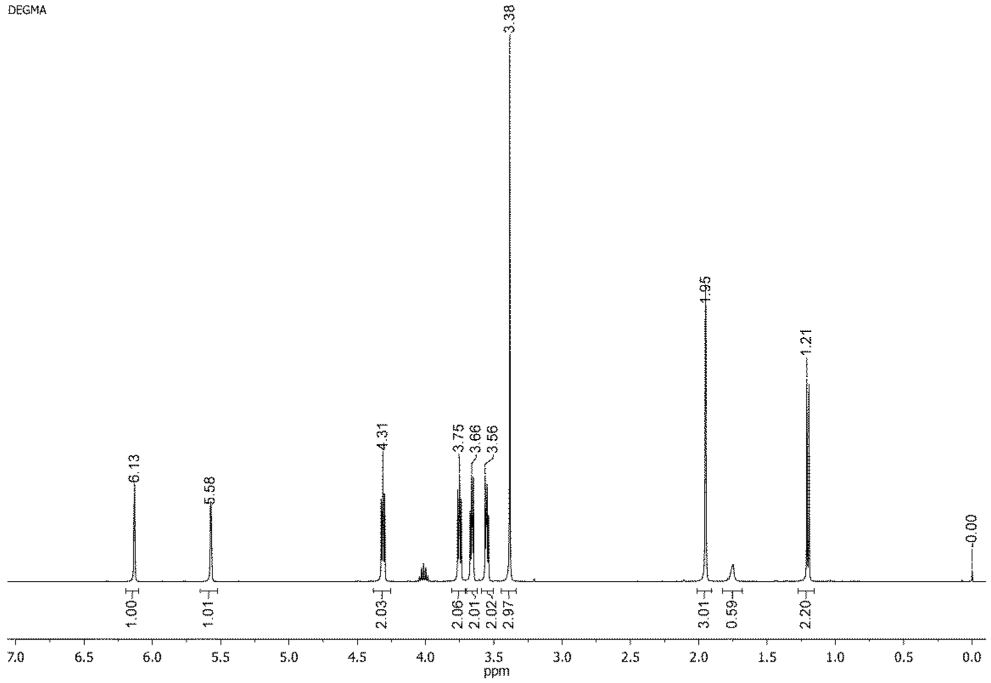

Figure S1. ^{1}H NMR ($CDCl_3$, 400 MHz) of diethylene glycol methyl ether methacrylate (DEGMA).

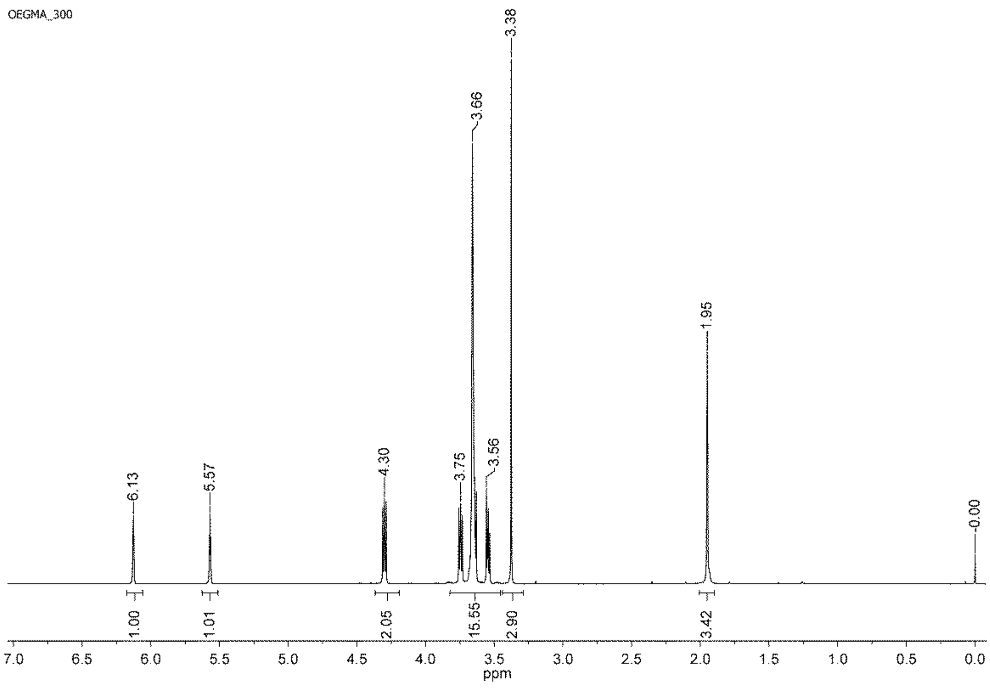

Figure S2. ^{1}H NMR ($CDCl_3$, 400 MHz) of oligoethylene glycol methyl ether methacrylate (OEGMA).

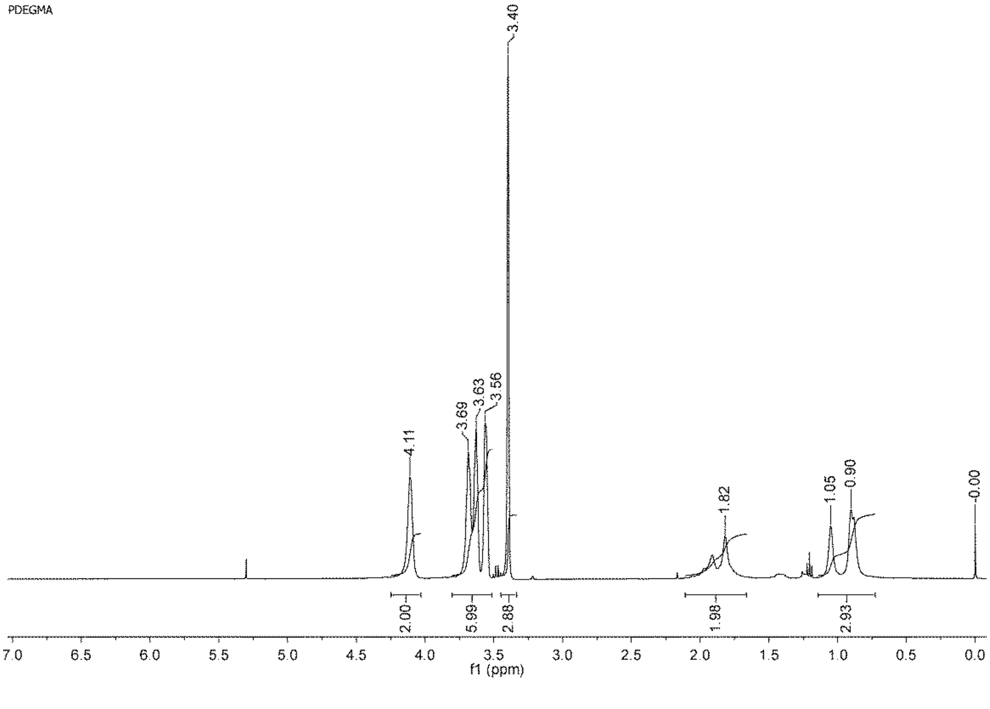

Figure S3. ^{1}H NMR ($CDCl_3$, 400 MHz) of poly(diethylene glycol methyl ether methacrylate (PDEGMA).

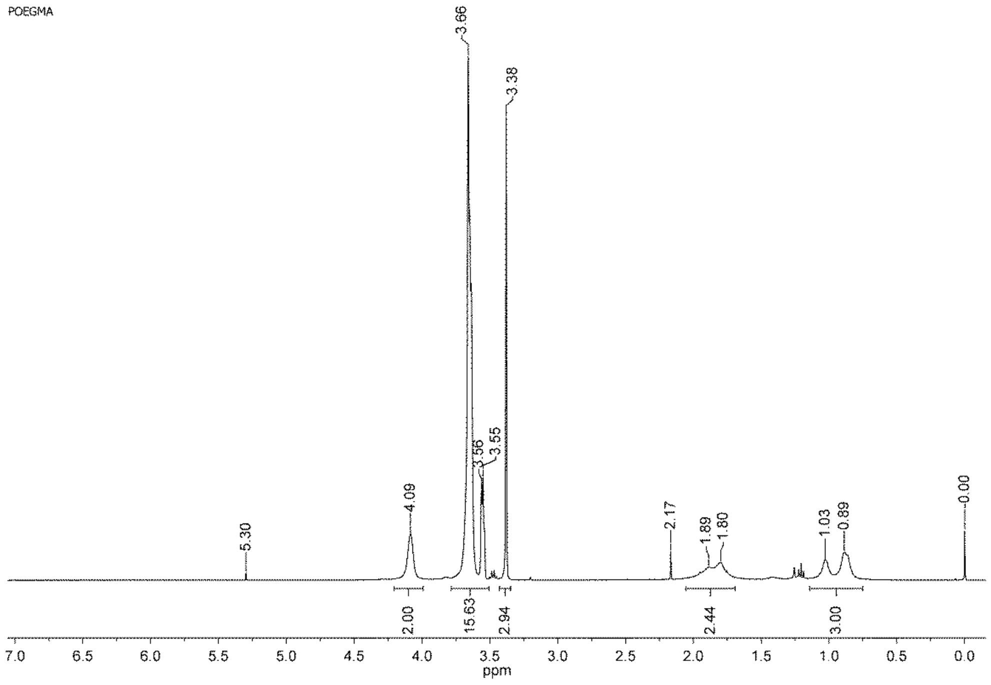

Figure S4. ^{1}H NMR ($CDCl_3$, 400 MHz) of diethylene glycol methyl ether methacrylate (POEGMA).

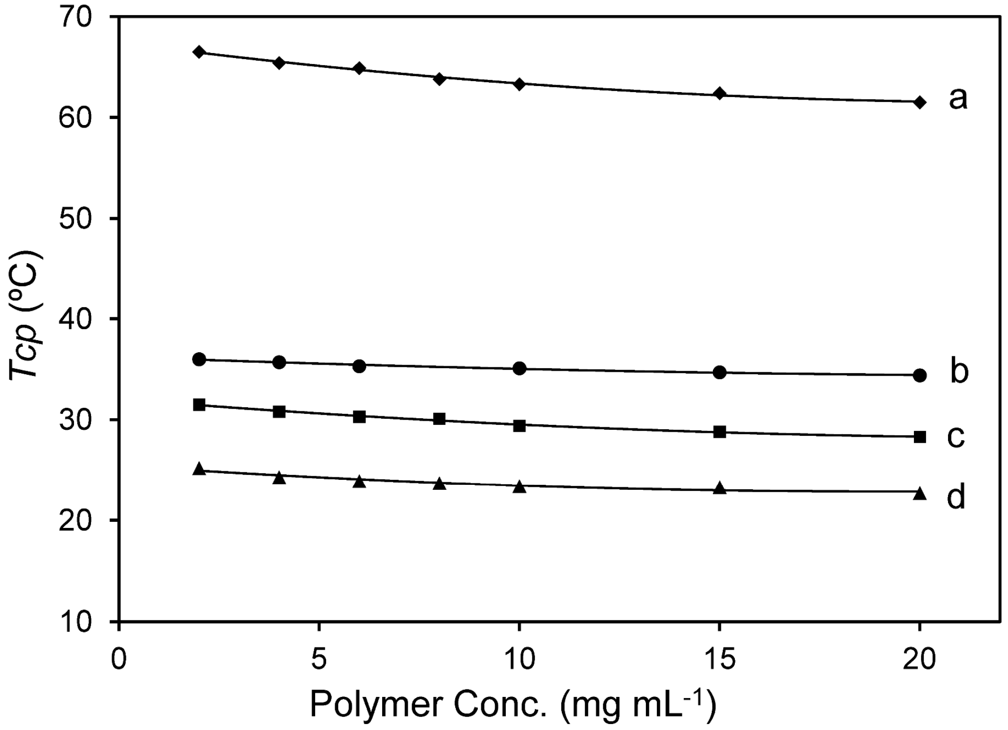

Figure S5. *Tcp* as a function of polymer concentration in water of a) POEGMA, b) poly(DEGMA-OEGMA) 75:25 mole ratio, c) poly(DEGMA-OEGMA) 86:14 mole ratio and d) PDEGMA.
